# Editorial: Neurogenesis and Gliogenesis as Potential Contributors to Neurorepair After Brain Damage

**DOI:** 10.3389/fnins.2022.852729

**Published:** 2022-02-15

**Authors:** Angélica Zepeda, Juan Manuel Encinas-Pérez, Noelia Urbán

**Affiliations:** ^1^Department for Angelica Zepeda, Instituto de Investigaciones Biomédicas, Universidad Nacional Autónoma de México, Mexico City, Mexico; ^2^Laboratory of Neural Stem Cells and Neurogenesis, Achucarro Basque Center for Neuroscience, Leioa, Spain; ^3^Departamento de Medicina Genómica y Toxicología Ambiental, Institute of Molecular Biotechnology of the Austrian Academy of Sciences (IMBA), Vienna Biocenter Campus (VBC), Vienna, Austria

**Keywords:** neuronal and glial lineage, epilepsy, excitotoxicity, neuroinflammation, functional recovery, spinal cord injury, memory, demyelination

The last 2 years were very challenging worldwide, not only for science, but for many aspects of daily life. We launched this Research Topic on December 2019 just days before Coivid-19 was first acknowledged to be of concern to the world. Many research labs around the globe were soon urged to maintain activities at the minimum, running experiments had to come to a sudden halt and we had to learn to communicate at distance while advancing our research projects. Despite the difficult circumstances, our contributors stayed commited with their purpose and successful research prevailed.

This Research Topic collects a body of original and review works on different aspects of the neurogenic process, as well as on the plasticity of neural progenitor and glial cells in physiological and pathological states. Of special interest is the capacity of the neural stem cells (NSCs) responsible of neurogenesis to also generate astroglial cells, a capability that in fact seems to be facilitated by some pathologial conditions.

Ceanga et al. review the literature supporting the beneficial vs. maladaptive impact of neurogenesis in the dentate gyrus (DG) of the hippocampus as well as of the neurogenic process taking place in the subventricular zone (SVZ), including the migration of newly born neurons toward the striatum as a result of experimentally induced stroke. The authors further discuss the effects of motor activity and enriched environment in functional recovery and refer to the response in the ipsi as well as in the contralateral area of the lesion.

Along this same line of research, Moura et al. show that neuronal hyperexcitation after the unilateral intrahippocampal administration of kainic acid (KA) or pilocarpine leads to the generation of cells derived from doublecortin progenitors. The authors further show that the cell fate of the newly generated cells differs depending on the injected drug. Thus, while pilocarpine favors a neuronal fate in the ipsi as well as in the contralateral hemisphere, KA favors neuronal differentiation only in the contralateral hemipshere, while inducing astrogliogenesis from doublecortin derived progenitors in the ipsilateral one. The authors also show that in the ipsilateral hemisphere injected with KA, a subset of cells derived from doublecortin progenitors display astroglial markers along with a radial glia morphology, and suggest that under hyperexcitation conditions, some cells could regress to a multipotent state. This work highlights the plastic response of NSCs and provides insights into how two epileptogenic drugs with different modes of action and potentially distinct effects in the GABAergic plexus of the DG, trigger differential responses from NSCs and neural progenitors.

In a similar line of research, Valcárcel-Martín et al. show that in a mouse model where DG NSCs express lysophosphatidic acid receptor 1 (LPA_1_)-GFP, the intrahippocampal injection of KA induces NSCs transformation into a reactive intermediate NSCs (React-NSCs) that are transitorily different from normal NSCs and from reactive astrocytes.

The above described work in turn gives a hint on the potential role of lipids in the response to brain damage, as reported by Zhou et al. In their work, the authors use cuprizone to provoke overall brain demyelination, along cognitive impairment. Lipid composition in several areas of the brain was affected due to the exposure to cuprizone. Notably, repetitve treatment with transcranial magnetic stimulation partially restored lipid composition and cognitive impairment, suggesting a potential link between both events.

Although recent progress has been made regarding the potency and plasticity of progenitors in the neurogenic niches (see Valcárcel-Martín et al. in this issue) it remains paramount to identify specific markers that distinguish NSCs from astrocytes. In this regard, Beyer et al. asked whether the expression of the astrocyte-specific gene Aldh1l1, could be used for lineage tracing in the subependymal zone (SEZ) and in the DG. The authors show that most astrocytes were specifically labeled in the DG, but in the SEZ, ependymal, and NSCs were labeled together with astrocytes. Their work highlights the important differences between both niches and provides the community with an important tool to study niche astrocytes in the DG.

Spinal cord injury leads to a decrease in hippocampal neurogenesis, as reviewed by Sefiani and Geoffroy. The authors point out at inflammation as a hallmark of spinal cord injury and discuss how inflammation can modulate the neurogenic process. Moreover, they discuss the prevalence of pathological aging in spinal cord injured patients and revise the relation between neurogenic decrease and cognitive decline, which often occurs in spinal cord injured patients. Ultimately, they discuss therapeutic options which aim at mitigating the effects of spinal cord injury while preserving hippocampal neurogenesis.

Overall, the functional evaluation of new neurons is key for unveiling their role in physiological as well as in pathological states and even more, for understanding their potential contribution in the process of brain repair. Hernández-Mercado and Zepeda review two widely used tasks, Morris water maze and contextual fear conditioning and memory as the prevailing protocols used to evaluate the functional role of hippocampal adult-born neurons. The authors emphasize the need to include procedures targeted at assessing pattern-separation and flexibility when using these tasks, as newly born neurons in the dentate gyrus have been shown to mediate these hippocampus-dependent cognitive functions. Throughout the review, the authors provide suggestions for the implementation of these tasks, as well as for standarizing different configuration features in order to have a common ground for the interpretation of results.

Altogether these studies showcase the interactive response of NSCs and neural progenitors, normally responsible for neurogenesis in the neurogenic niches, but also highlight their gliogenic capability. This is an emerging field whose relevance rises in pathological conditions. We are in the first stages of understanding whether a shift in the neurogenic/gliogenic balance in conditions such as stroke or epilepsy is beneficial or detrimental. The neurogenic/gliogenic balance is a prospect therapeutic target and hopefully further research will enable to develop tools to unveil the impact of each event as well as to undercover the mechanisms involved in each process.

## Author Contributions

All authors listed have made a substantial, direct, and intellectual contribution to the work and approved it for publication.

## Funding

AZ was supported by Programa de Apoyos para la Superación del Personal Académico de la UNAM (PASPA)-Dirección General de Asuntos del Personal Académico (DGAPA)-UNAM during a sabbatical stay. NU was supported by IMBA and by grants from the Austrian Science Fund (FWF: SFB-78, SFB-79, and Docfunds-Scorpion and Docfunds-Smich). JMEP was supported by grant PID2019-104766RB-C21 (Spanish Ministry of Innovation and Science-MICINN) and Apoyo Dravet.

## Conflict of Interest

The authors declare that the research was conducted in the absence of any commercial or financial relationships that could be construed as a potential conflict of interest.

## Publisher's Note

All claims expressed in this article are solely those of the authors and do not necessarily represent those of their affiliated organizations, or those of the publisher, the editors and the reviewers. Any product that may be evaluated in this article, or claim that may be made by its manufacturer, is not guaranteed or endorsed by the publisher.

